# Serum metabolomic profiling reveals LysoPC/PC depletion as a potential biomarker to detect avian reoviral infection in neonatal broiler chickens

**DOI:** 10.3389/fcimb.2026.1750590

**Published:** 2026-05-08

**Authors:** Asha Ranaraja, Iresha Subhasinghe, Noor Ahmad Shaik, Babajan Banaganapalli, Shelly Popowich, Lisanework E. Ayalew, Rupasri Mandal, David S. Wishart, Suresh Tikoo, Susantha Gomis

**Affiliations:** 1 Department of Veterinary Pathology, Western College of Veterinary Medicine, University of Saskatchewan, Saskatoon, SK, Canada; 2 Department of Genetic Medicine, Faculty of Medicine, King Abdulaziz University, Jeddah, Saudi Arabia; 3 Department of Pathology and Microbiology, Atlantic Veterinary College, University of Prince Edward Island, Charlottetown, PE, Canada; 4 Departments of Biological Sciences and Computing Science, University of Alberta, Edmonton, AB, Canada; 5 Vaccinology and Immunotherapy, School of Public Health, University of Saskatchewan, Saskatoon, SK, Canada

**Keywords:** avian reovirus, broiler chicken, LysoPC/PC, serum metabolomics, viral pathogenesis

## Abstract

**Introduction:**

Avian reovirus (ARV) infection causes viral arthritis in broiler chickens, leading to lameness and substantial economic loss in poultry production. Current conventional diagnostic methods detect infection only after clinical signs appear, limiting the ability to prevent disease progression. Hence, this study utilized serum metabolomics to identify early biomarkers of ARV infection prior to tissue damage.

**Methods:**

A total of 150 serum samples were collected from ARV-challenged broilers at 24, 48, and 72 h post-infection, along with uninfected controls. The samples underwent liquid chromatography–tandem mass spectrometry (LC–MS/ MS) profiling, followed by preprocessing through standardization, log2 transformation, and batch correction. Differential metabolite expressions were assessed using *limma*, and pathway enrichment was performed by using hypergeometric testing. Logistic regression (LR) and receiver operating characteristic (AUC) analyses identified metabolites that were consistently altered across all timepoints.

**Results:**

Among 587 metabolites detected, 371, 96, and 402 were differentially expressed at 24, 48, and 72 h, respectively. Our findings have shown that lipid biomarkers like lysophosphatidylcholine (LysoPC) a C14:0, LysoPC a C20:4, and phosphatidylcholine (PC) aa C38:5 were consistently decreased and demonstrated strong single-analyte discrimination, with LysoPC a C14:0 showing nearperfect specificity at 24/72 h (AUC ≈ 1.00). Conversely, the levels of non-lipids like N-acetylputrescine (NAP) and 2-hydroxyglutarate (2-HG) were elevated, exhibiting high AUC (≈0.97–1.00) as sensitive early-screening markers. The pathway analysis revealed significant alterations in arginine–proline, branched-chain amino acid, glycine/serine/threonine, and one-carbon/folate metabolism.

**Discussion:**

These findings demonstrated that ARV infection induced early metabolic reprogramming within 24 h, with lipid remodeling and polyamine catabolism identified as key early biomarkers. Serum metabolomics thus provides a rapid, non-invasive tool for the early detection and surveillance of ARV infection in poultry flocks.

## 1 Introduction

Arthritis or tenosynovitis induced by avian reovirus (ARV) in broilers is an economically significant disease of poultry. The disease was first described in 1959 (Olson, 1959), and later the causative agent was identified as ARV (Olson and Weiss, 1972; Walker et al., 1972). The clinical representation includes unilateral or bilateral inflammation of the hock joint, resulting in lameness. Furthermore, it leads to poor growth and production and, sometimes death, causing considerable economic losses. The virus has also been associated with other syndromes, including runting/stunting, malabsorption syndrome, respiratory disease, myocarditis, and hepatitis (Jones, 2000), although ARV is not always pathogenic and has been reported from routine examinations in apparently healthy poultry flocks. However, chickens are more susceptible to ARV during the growing/rearing period and become increasingly resistant to infection with age. Morbidity is variable and can reach up to 100%, while mortality is low, generally <6% in the case of this viral infection (Saif, 2009).

ARV is classified under the *Reoviridae* family in the genus *Orthoreovirus*, a double-stranded, non-enveloped RNA virus with the capsid composed of two icosahedrally concentric protein shells, with the size ranging between 70 and 80 nm in diameter (Benavente and Martínez-Costas, 2007; Spandidos and Graham, 1976). Based on the most variable cell attachment, ARV is classified into six genotypic cluster groups based on the most variable cell attachment Sigma-C (σC) protein (Liu et al., 2003; Ayalew et al., 2017). The protein is 326 amino acids in length, is encoded by the tricistronic S1 genome segment, and contains antibody-neutralizing specific epitopes. Moreover, they are resistant to heat, proteolytic enzymes, various disinfectants, and a wide spectrum of pH (Calvo et al., 2005; Wickramasinghe et al., 1993).

In the past decade, ARV caused considerable economic losses and animal welfare issues in the poultry industry in Canada and USA, with a drastic increase in ARV-associated outbreaks with emerging variants (Ayalew et al., 2017; Palomino-Tapia et al., 2018). Previous studies have demonstrated that the emerging virulent circulating strains were genetically diverse and evolutionarily distant from the vaccine and vaccine-related field strains. Moreover, several reports support the evidence that the emerging ARVs can break vaccine-induced immunity, resulting in the persistence of ARV-associated disease in poultry flocks (Liu et al., 2003; Goldenberg et al., 2010). The poultry industry in North America uses autogenous vaccines as alternatives to control and prevent the ARV-associated disease due to the absence of a commercial vaccine against the currently circulating emerging ARV variants. The effectiveness of autogenous vaccines can be compounded by several factors, including the co-circulation of multiple antigenic variants in the farm/region and the lack of robust characterization of circulating variants and antigen inclusion criteria (Gamble and Sellers, 2022).

The early detection of ARV is crucial to prevent economic losses in controlling pathogens and thus prevent imminent disease outbreaks. The chicken industry is currently relying on the diagnosis of ARV through methods that include virus isolation, molecular detection (e.g., RT-PCR), serological assays like ELISA, and histopathology. However, serological tests detect diseases only 5 to 6 days after pathogenic exposure. While metabolic alterations have been observed in other viral diseases (e.g., infectious bursal disease), achieving a diagnosis within 24 h post-infection, before clinical signs appear, remains a significant challenge (Wang et al., 2024). Accordingly, early disease detection through metabolite alterations has emerged as a promising approach to improve disease prevention and management practices in poultry. The objective of this study was to identify the early serum metabolic biomarkers of ARV infection in broiler chickens.

## 2 Materials and methods

### 2.1 Housing and maintenance of chickens

The experiment protocol was authorized by the University of Saskatchewan Animal Research Ethics Board (protocol #20160010). All methods were performed in accordance with the guidelines and regulations of the Canadian Council on Animal Care. Euthanasia was performed by cervical dislocation. Mixed-sex (male and female) day-old Ross commercial broiler chicks were obtained from a hatchery in Saskatchewan and assigned to experimental groups in animal isolation rooms at the University of Saskatchewan’s Western College of Veterinary Medicine’s Animal Care Unit. The control and treatment birds were placed in separate rooms. The temperature in the broiler chick rooms was regulated. During their first week of life, the temperature was set to 32 °C. Thereafter, the temperature was lowered daily by 0.5 °C until a temperature of 20 °C is achieved. Feeding, air pressure variations, light levels, and stringent hygienic conditions were maintained per Aviagen’s guidelines. Water and commercial starter feed containing 20% crude protein were given *ad libitum*. A HEPA filter was used to exhaust the air in the chick isolation room and supply new intake air at 15 to 20 air changes per hour without air circulation. For the first 2 days after hatching, constant light was provided at 30 lx for 24 h a day. Thereafter, the lux and duration were decreased until 10–20 lx and 7 h of darkness were achieved.

### 2.2 ARV animal model

The ARV challenge model used for this study was previously established in our laboratory using field isolates from ARV-positive broiler farms in Saskatchewan that exhibited clinical signs of tenosynovitis and lameness (Ayalew et al., 2022). Briefly, the tendons were homogenized and centrifuged, and the supernatant was filtered and then added to leghorn male hepatoma (LMH) cells to observe the appearance of cytopathic effects (CPE). The isolated reovirus displayed CPE in cell culture, including syncytia formation and cell fusion. The observations were interpreted as previously described (Ayalew et al., 2022). The virus was subsequently propagated in culture media, and purified, and whole-genome sequences for genotypic cluster (C) groups CII, CIV, CV, and CVI were obtained and stored at -80°C for challenge studies. For the challenge, the broiler chickens were injected with 100 µL of 1 × 10^5^ plaque-forming units (PFU)/mL of ARV into the left footpad (Ayalew et al., 2022).

Two separate experiments were conducted to assess changes in the metabolomic profile of broiler chickens at different timepoints following the challenges with avian reovirus.

### 2.3 Experiment 1

On the day of hatch, a total of 90 broiler chicks were allocated into two experimental groups [control group (*n* = 30) and treatment groups (*n* = 60)], representing a convenience sampling approach. At 7 days of age, the treatment group of birds was challenged in the left foot pad with 1 × 10^5^ (PFU)/mL of CII avian reovirus in a volume of 100 µL per bird using the previously established reovirus animal model (Ayalew et al., 2022). The control group was composed of uninfected birds and was given 100 µL of saline per bird in the left footpad. Following the challenge, the birds were closely monitored for swelling in the footpads, hock joints, and lameness. At 24 h post-challenge, blood samples were collected from the unchallenged control group (*n* = 30) and the ARV-challenged group (*n* = 30) into serum tubes. A total of 30 birds were sampled again for blood at 72 h post-ARV challenge. At each point, all birds that had been sampled for blood were humanely euthanized by cervical dislocation and examined post-mortem for gross lesions.

### 2.4 Experiment 2

The second experiment consisted of two groups of birds, the control (*n* = 30) and the treatment groups (*n* = 30). The birds were challenged with genotypic cluster CVI ARV at 12 days of age, following the same challenge model as in the first experiment (Ayalew et al., 2022). The challenge day, ARV cluster, and sampling timepoint were altered by considering the variability of infection and timing in the field conditions. The control group was given saline in the left footpad.

The challenged birds were observed for footpad and hock joint swelling and lameness. Blood samples were taken into serum tubes at 48 h post-infection. Simultaneously, the gastrocnemius tendon was sampled from five birds (*n* = 5) per group (control and ARV-infected birds) into 10% neutral-buffered formalin for histopathological examination and later stained with hematoxylin and eosin stain to observe inflammatory changes. Histopathological scoring of tendons was performed by an experienced avian pathologist as previously described (Ayalew et al., 2023), namely: score 0 = normal, score 1 = mild tendinitis, score 2 = moderate tendinitis, and score 3 = severe tendinitis. The birds were monitored for 7 days post-ARV challenge, and the body weight of all birds (control and infected) was measured at the end of the experiment. An independent two-sample *t*-test (Welch’s *t*-test) was performed to compare the mean body weights of control and infected birds, accounting for unequal variances between groups.

### 2.5 Separation of serum and preparation of samples for metabolomic analysis

Immediately after blood collection from the control (*n* = 30) and ARV-challenged groups (*n* = 30), the serum tubes were centrifuged at 1,000 *g* for 10 min, using a Clay Adams serofuge 2002 (Becton Dickinson, USA) to separate the serum. Then, the serum was aliquoted into 1.5-mL microcentrifuge tubes, flash-frozen in dry ice, and stored at -80 °C until further metabolomic analysis. All of the samples were submitted for analysis using liquid chromatography–mass spectrometry (LC–MS) to the Metabolomic Innovation Center (TMIC), in Alberta, Canada, according to the recommended metabolomic sample submission guidelines.

### 2.6 Metabolomics assay

A metabolomics analysis of the serum samples was performed to quantify 900 endogenous metabolites like amino acids, sugars, organic acids, nucleobases, vitamins, and lipids (e.g., sphingomyelins, triglycerides). This assay was conducted on TMIC Mega Metabolomics assay, which employs a dual approach combining direct injection (DI) mass spectrometry and reverse-phase LC–MS/MS.

Isotope-labeled internal standards (ISTDs) at concentrations between 1 and 10 µM, along with chemical derivatizing agents such as phenyl isothiocyanate (PITC) for amino acids and 3-nitrophenylhydrazine (3-NPH) for organic acids, were introduced to enhance ionization and separation during mass spectrometry. The stock solutions were initially prepared at concentrations ranging from 0.01 to 1 mM and were further diluted to create calibration standards (Cal1–Cal7) and quality control (QC) standards at low, medium, and high levels (0.05, 0.5, and 5 µM, respectively). For amino acids, amino acid derivatives, biogenic amines, and nucleotides/nucleosides, PITC derivatives were performed by drying the samples under a nitrogen stream, adding a 5% PITC derivatization solution, and extracting the targeted analytes using methanol containing 5 mM ammonium acetate.

For the LC–MS/MS analysis, 50 µL of the extract was combined with 450 µL of LC–MS water. In contrast, for the direct infusion tandem mass spectrometry (DI-MS/MS) analysis, 10 µL of the extract was combined with 490 µL of direct flow injection buffer for lipid and acylcarnitine profiling. Three QC samples at varying concentrations were analyzed in triplicate to ensure precision, and blank samples were included to evaluate possible contamination. Data normalization was performed using isotope-labeled internal standards included in the TMIC assay, enabling correction for analytical variability and ionization efficiency. Limits of detection (LOD) and quantification (LOQ) for each metabolite were established using calibration curves generated from standard mixtures as per TMIC protocols. Missing values were imputed using half of the metabolite-specific LOD to account for values below the detection limit. The assay results were provided in CSV/XLSX format, comprising raw metabolite intensities, internal-standard-adjusted concentrations, retention times, mass-to-charge (*m*/*z*) ratios, peak areas, and normalized concentrations.

### 2.7 Bioinformatics analysis of metabolomics data

The metabolomics dataset, containing metabolite concentrations expressed in micromolar (µM) units, was analyzed using a structured workflow to ensure data quality, consistency, and reproducibility across experimental groups. The study included control and ARV-challenged samples collected at 24, 48, and 72 h. Experiment 1 comprised samples from 24 and 72 h, whereas experiment 2 included samples from 48 h, resulting in a total of 150 samples (*n* = 30 per timepoint). The analytical workflow included sequential steps of preprocessing, normalization, batch correction, differential analysis, pathway interpretation, and biomarker evaluation.

#### 2.7.1 Data preprocessing

Raw data handling and preprocessing were conducted in Python (v3.x) using the pandas and numpy libraries for data curation and formatting. Metabolites with constant values or with ≥75% missing data across samples were excluded. The remaining missing values, considered to represent concentrations below the detection limit, were imputed using half of the metabolite-specific limit of detection (LOD) as defined during TMIC-based quantification.

To minimize systematic variation, data were normalized using median scaling, whereby each metabolite value was divided by the median value of that metabolite across all samples (van den Berg et al., 2006). The normalized dataset was then log_2_-transformed to stabilize the variance and reduce the influence of extreme values, followed by mean centering and standardization to ensure comparability across metabolites.

#### 2.7.2 Statistical analysis

Subsequent analyses were conducted in R statistical software. Batch effects arising from the two experimental runs were corrected using the sva package. Principal component analysis (PCA) was used to evaluate the data structure before and after batch correction.

Differential metabolite analysis was performed using the limma package, applying linear models with empirical Bayes moderation. Comparisons were made between control and ARV-infected groups at each timepoint (24, 48, and 72 h). Metabolites with an adjusted *p*-value <0.05 and absolute fold change >1.5 were considered significantly altered.

#### 2.7.3 Pathway and biomarker analysis

To assess the biological significance, pathway enrichment analysis of significantly altered metabolites was performed using MetaboAnalyst, integrating hypergeometric testing with pathway topology analysis, with a significance threshold of *p <*0.05.

To further identify clinically relevant features, biomarker discovery was performed using logistic regression (LR) to identify metabolites that were consistently associated with infection across all timepoints. The predictive performance of the selected biomarkers was evaluated using receiver operating characteristic (ROC) analysis. Standardized log_2_ values were back-transformed to micromolar using the equation µM = 2^(μ + σ × value) − ϵ, where μ represents the location parameter on the log_2_ scale, σ the standard deviation, and ϵ the pseudo-count. ROC curves (sensitivity vs. 1 − specificity) were generated using the pROC R package, and the area under the curve (AUC) with 95% confidence intervals was estimated using DeLong’s method. Direction-specific classification rules were applied (downregulated: µM ≤ cutoff; upregulated: µM ≥ cutoff), and optimal thresholds were determined using Youden’s index, with the corresponding sensitivity and specificity reported.

#### 2.7.4 Data visualization and network analysis

All statistical analyses and visualizations were primarily performed in R statistical software (https://www.r-project.org/). The sva package (https://bioconductor.org/packages/release/bioc/html/sva.html) was used for batch correction and PCA-based assessment of batch effects, while the limma package (https://bioconductor.org/packages/release/bioc/html/limma.html) was used for differential metabolite analysis. Data visualization, including boxplots, violin plots, and PCA plots, was performed using ggplot2 (https://ggplot2.tidyverse.org/), and volcano plots were generated using EnhancedVolcano (https://github.com/kevinblighe/EnhancedVolcano). Pathway enrichment analysis was conducted using MetaboAnalyst (https://www.metaboanalyst.ca/), metabolite processing was supported by XCMS (https://bioconductor.org/packages/release/bioc/html/xcms.html), and network visualization was performed using Cytoscape (https://cytoscape.org/).

## 3 Results

### 3.1 Clinical and histopathological outcome

**Figure 1:**
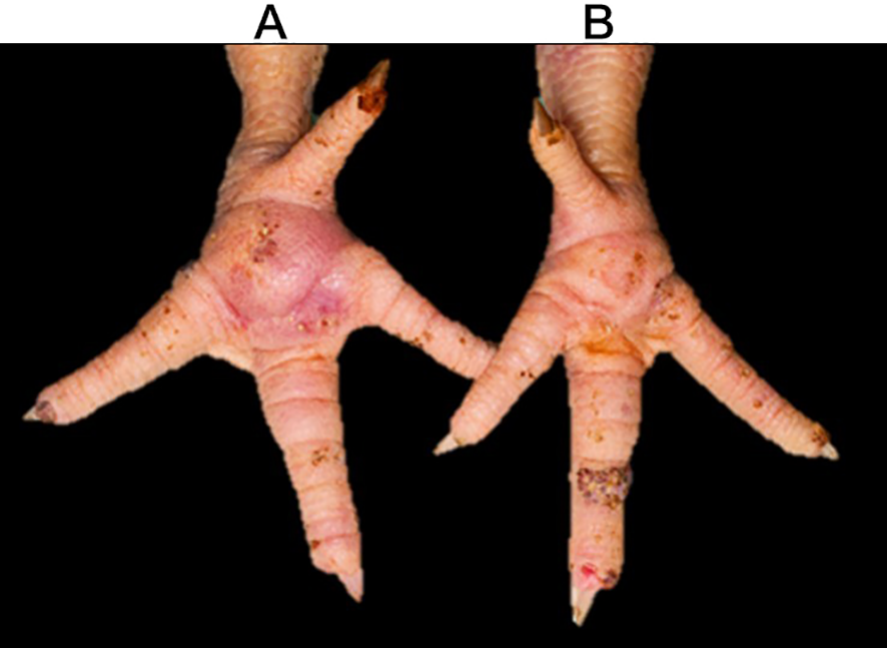
ARV infection signs in broiler chickens **(A)** Moderate–severe footpad lesion on the left footpad in an ARV broiler bird at 72 h post-infection. **(B)** Non-challenged right foot pad of the same bird at 72 h.

In experiment 1, no foot pad swelling or lameness was observed in the birds challenged with ARV 24 h post-challenge. However, at 72 h post-challenge, 96.7% of the birds developed moderate to severe footpad lesions (**Figure 1A, B**) and were lame, while 3.3% had mild to moderate footpad lesions based on gross footpad scoring (Ayalew et al., 2023). There were no mortalities after the challenge.

**Figure 2:**
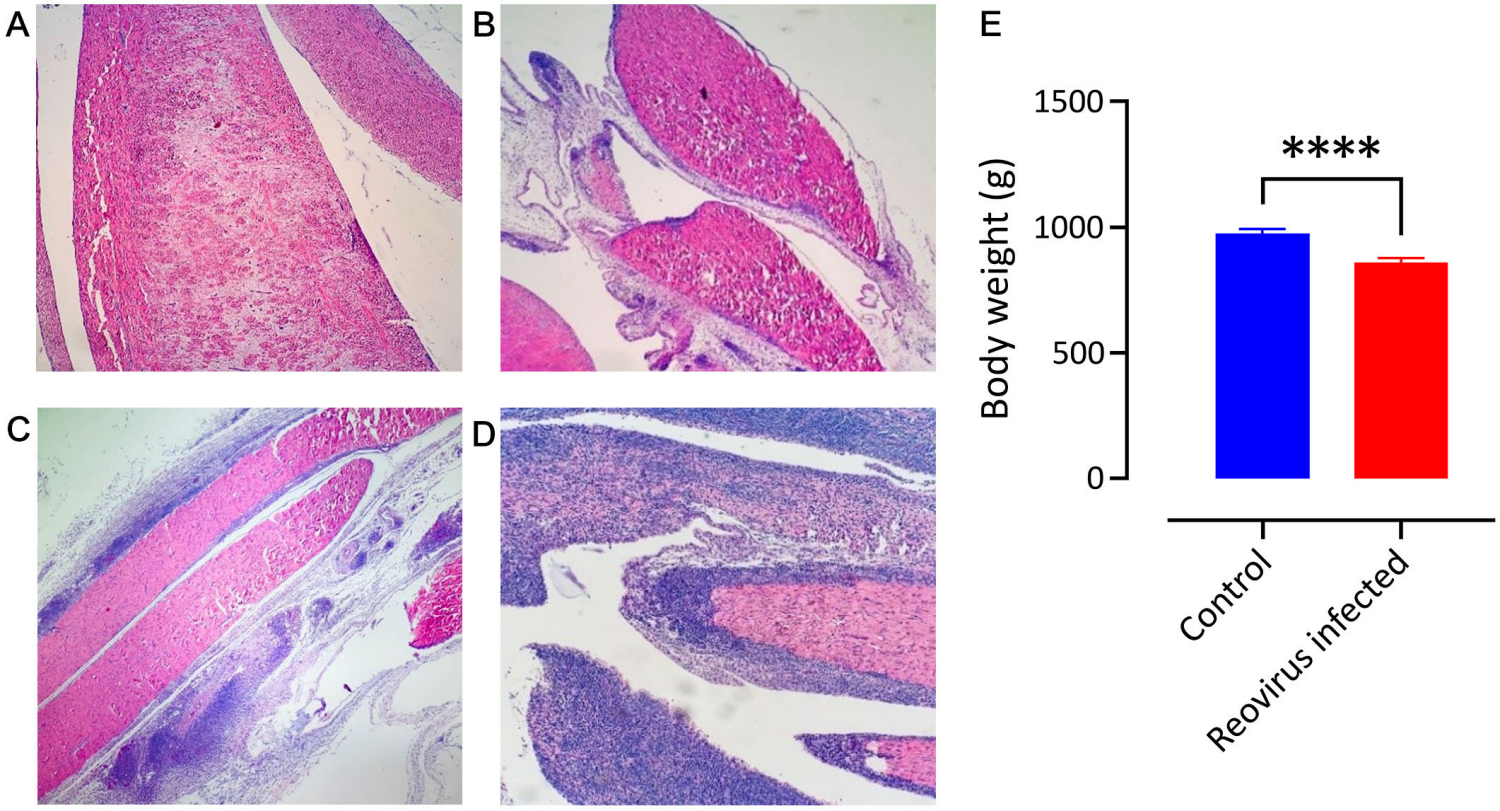
Histopathological lesions of gastrocnemius tendon 48 h post-ARV challenge. Hematoxylin-and-eosin-stained gastrocnemius tendon of infected birds showing clusters of lymphocyte infiltration in the tendon sheath (×4 magnification). **(A)** Normal gastrocnemius tendon (score 0), **(B)** mild infiltration of lymphocytes in the tendon sheath (score 1), **(C)** moderate lymphocyte infiltration in the tendon sheath (score 2), and **(D)** severe lymphocyte infiltration with few heterophils in the tendon sheath (score 3). **(E)** A comparison of the mean body weight of the ARV-infected and control birds shows a significant impairment of weight gain in the infected birds (****p < 0.0001).

In experiment 2, none of the ARV-challenged birds showed mortality at 48 h post-infection. However, 90% of the infected birds showed mild foot pad swelling without lameness. During the histopathological examination of the gastrocnemius tendons of the five birds sampled, varying degrees of lymphocyte infiltration were shown following ARV challenge, ranging from normal tendons (20%), tendons with mild lymphocyte infiltration (20%), tendons with moderate lymphocyte infiltration (40%), and tendons with severe lymphocyte infiltration with a few heterophils (20%) as previously described in this animal model of ARV (Ayalew et al., 2023) (**Figures 2A–D**). The comparison of the mean body weights of the ARV-infected birds (*n* = 30) with uninfected birds (*n* = 30) at 7 days post-challenge showed a statistically significant weight reduction in the infected group when compared to the control (*p* < 0.0001) (**Figure 2E**).

### 3.2 Data normalization and batch effects correction

A total of 150 serum samples, collected across two experimental batches representing controls and ARV-challenged groups at 24, 48, and 72 h, were subjected to metabolomics profiling. From 634 detected metabolites, 587 were retained after filtering out those with ≥75% missing or invariant values. Missing entries were imputed at half the metabolite-specific limit of detection (LOD). Raw intensity values displayed wide variability and skewed distributions (**Supplementary Table S1A**; **Supplementary Figure S1A**). Normalization using median scaling, log_2_ transformation, and z-standardization stabilized the variance and improved the comparability across samples, as confirmed by post-normalization boxplots with distributions centered near zero and the consistent variance across groups (**Supplementary Figure S1B**). The PCA of the uncorrected dataset revealed strong batch effects, with samples clustered by experimental batch rather than biological condition (PC1 = 40.6%, PC2 = 12.4%) (**Supplementary Figure S2A**). Following batch correction with the *sva* package, batch-related variation was minimized, and the samples were grouped according to biological status. In the corrected dataset, PC1 (27.7%) and PC2 (16.4%) captured biologically relevant separation, with ARV-infected birds, particularly at 72 h, clearly distinguished from controls (**Supplementary Figure S2B**). This confirms that batch correction preserved the infection-driven metabolic variation while reducing the confounding batch effects.

### 3.3 Distinct metabolic shifts observed at 24, 48, and 72 h post-infection

#### 3.3.1 Data at 24 h post-infection

**Figure 3:**
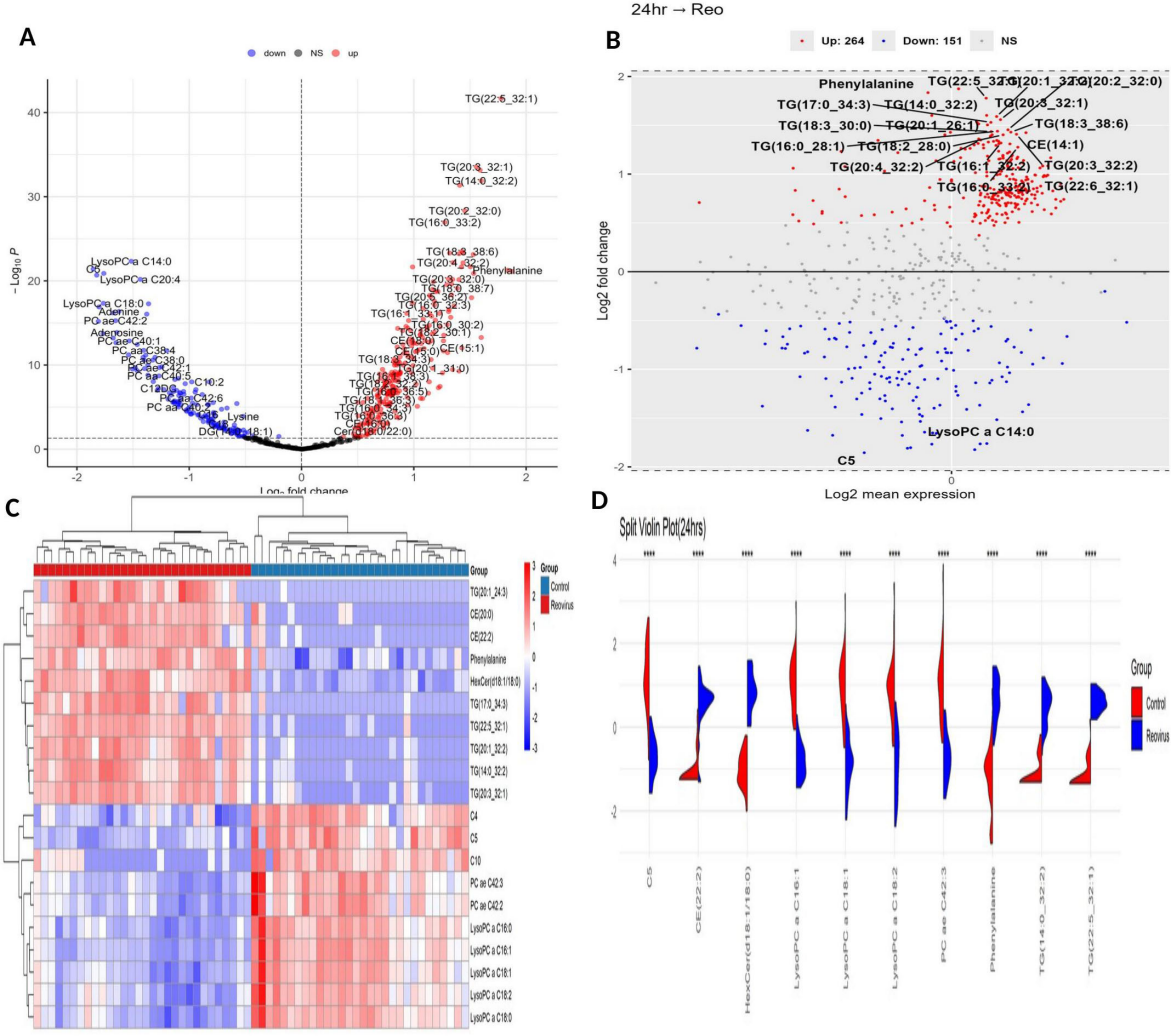
Metabolic alterations 24 h post-ARV infection. **(A)** Volcano plot of 371 significantly altered metabolites (238 upregulated, 133 downregulated; *P*_adj_ < 0.05, FC > |1.5|). **(B)** MA plot highlighting the upregulated neutral lipids and phenylalanine versus suppressed LysoPCs and acylcarnitines at low abundance. **(C)** Heatmap showing the distinct separation of metabolite expression among control and infected groups. **(D)** Violin plots validating the depletion of LysoPCs and PC ae C42:3 and the elevation of cholesteryl ester, triglyceride, and HexCer.

At 24 h post-infection, a total of 371 metabolites were identified as significantly differentially expressed (*P*_adj_ < 0.05, FC > |1.5|), comprising 238 upregulated (64.2%) and 133 downregulated (35.8%) features. The volcano plot (**Figure 3A**) illustrates this distribution, clearly delineating the upregulated and downregulated metabolite populations. These 371 differentially expressed metabolites are comprised of 304 lipid metabolites (82.0%) and 67 non-lipid metabolites (18.0%) spanning multiple chemical classes, such as triglycerides (*n* = 192), phosphatidylcholines (*n* = 55), amino acid derivatives (21), cholesteryl esters (*n* = 20), and sphingomyelins (*n* = 13).

To assess the overall distribution of differential expression, density histograms of log_2_ fold change (logFC) and mean expression (AveExpr) were generated (**Supplementary Figures S3A, B**, respectively). The upregulated metabolites showed a mean logFC of 0.96 ± 0.26, whereas the downregulated metabolites averaged –1.86 ± 0.33, indicating that although more metabolites were upregulated, the magnitude of suppression was stronger. This pattern suggests a virus-driven metabolic shift favoring selective downregulation. The logFC distribution (**Supplementary Figure S3A**) revealed a rightward shift for upregulated features and broader dispersion among downregulated ones, consistent with greater variability and intensity of suppression. The expression values were skewed toward lower abundance, with a long-tailed distribution reflecting a few highly expressed metabolites (**Supplementary Figure S3B**).

The MA plot (**Figure 3B**) further confirmed that most upregulated features, especially lipids, were of moderate expression, while suppressed features such as acylcarnitines and lysophosphatidylcholine (LysoPCs) clustered at lower abundance. The heatmap revealed distinct clustering between the control and infected groups. The upregulated clusters included neutral lipids [CE (22:2), CE (20:0), multiple TGs] and phenylalanine, while the downregulated features were mainly LysoPCs, PCs, and short chain acylcarnitines (C4, C5, C10) (**Figure 3C**). The violin plots further validate these findings, illustrating the distribution of the selected metabolites—for instance, LysoPC and PC ae C42:3 were elevated in the infected samples, while CE (22:2), triglyceride (TG) (22:5_32:1), and HexCer(d18:1/18:0) were reduced, matching the heatmap pattern (**Figure 3D**).

#### 3.3.2 Data at 48 h post-infection

**Figure 4:**
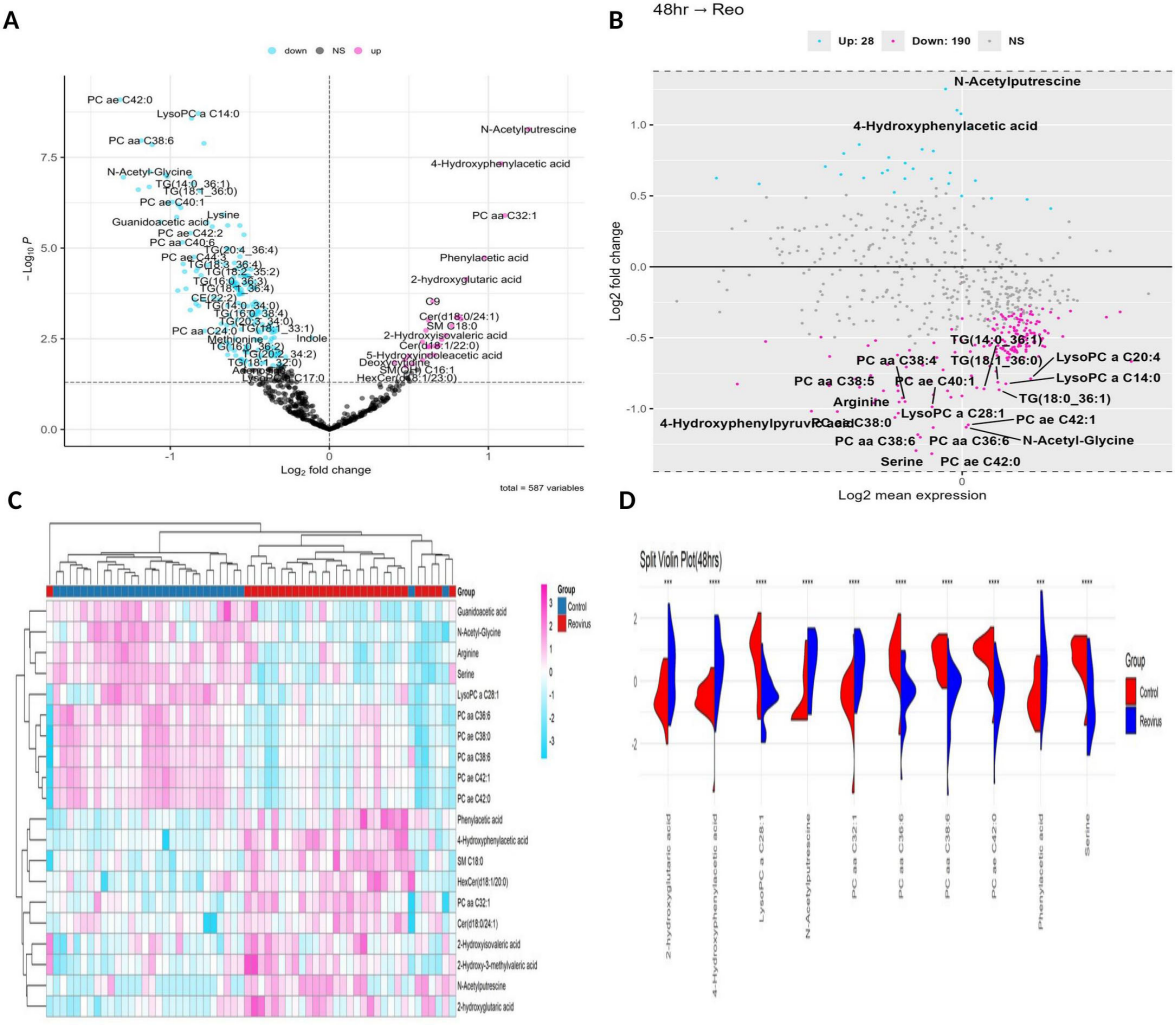
Metabolic alterations 48 h post ARV infection. **(A)** Volcano plot of 96 significantly altered metabolites (22 upregulated, 74 downregulated; *P*_adj_ < 0.05, FC > |1.5|). **(B)** MA plot illustrates the modest upregulation of amino/organic acids and consistent downregulation of phospholipids and triglycerides. **(C)** Heatmap showing the clear separation of control and infected groups, with lipid suppression and amino/organic acid elevation. **(D)** Split violin plots show the depletion of membrane lipids (e.g., LysoPCs) and the elevation of organic acids (e.g., N-acetylputrescine) in infected samples compared to controls.

The metabolomic profiling revealed 96 significantly dysregulated metabolites (*P*_adj_ < 0.05, FC > |1.5|), comprising 22 upregulated (22.9%) and 74 downregulated (77.1%) features. These 96 metabolites comprised 62 lipid metabolites (64.6%) and 34 non-lipid metabolites (35.4%). The mean fold change for upregulated metabolites was 0.77 ± 0.18, while the downregulated metabolites had a mean fold change of -0.79 ± 0.18, indicating moderate variability in metabolic shifts. The volcano plot highlights this differential distribution, with notable upregulation of N-acetylputrescine, PC aa C32:1, and 4-hydroxyphenylacetic acid, while lipids such as PC aa C42:0, TG (14:0_36:1), and LysoPC a C14:0 were prominently decreased (**Figure 4A**). The overall distribution of fold changes is shown in the log_2_FC histogram, which reveals a slight leftward skew toward downregulated metabolites and a tighter range of logFC values, reflecting a modest yet consistent suppression trend (**Supplementary Figure S3C**). In parallel, the average expression distribution confirms that most features were of low to moderate abundance, with a few highly expressed metabolites creating a long-tailed distribution (**Supplementary Figure S3D**). Further insight into the expression trends is captured in the MA plot, where metabolite log_2_FC is plotted against average expression (**Figure 4B**). In the MA plot, several phospholipids, lysophospholipid, and triglyceride species cluster toward negative log2 fold changes, whereas a smaller number of non-lipid metabolites appear on the positive side. Heatmap clustering of the significant metabolites shows a group-wise separation between infected and control samples (**Figure 4C**). This pattern is further illustrated by split-violin plots, which show distinct abundance distributions for representative metabolites between the two groups (**Figure 4D**).

#### 3.3.3 Data at 72 h post-infection

**Figure 5:**
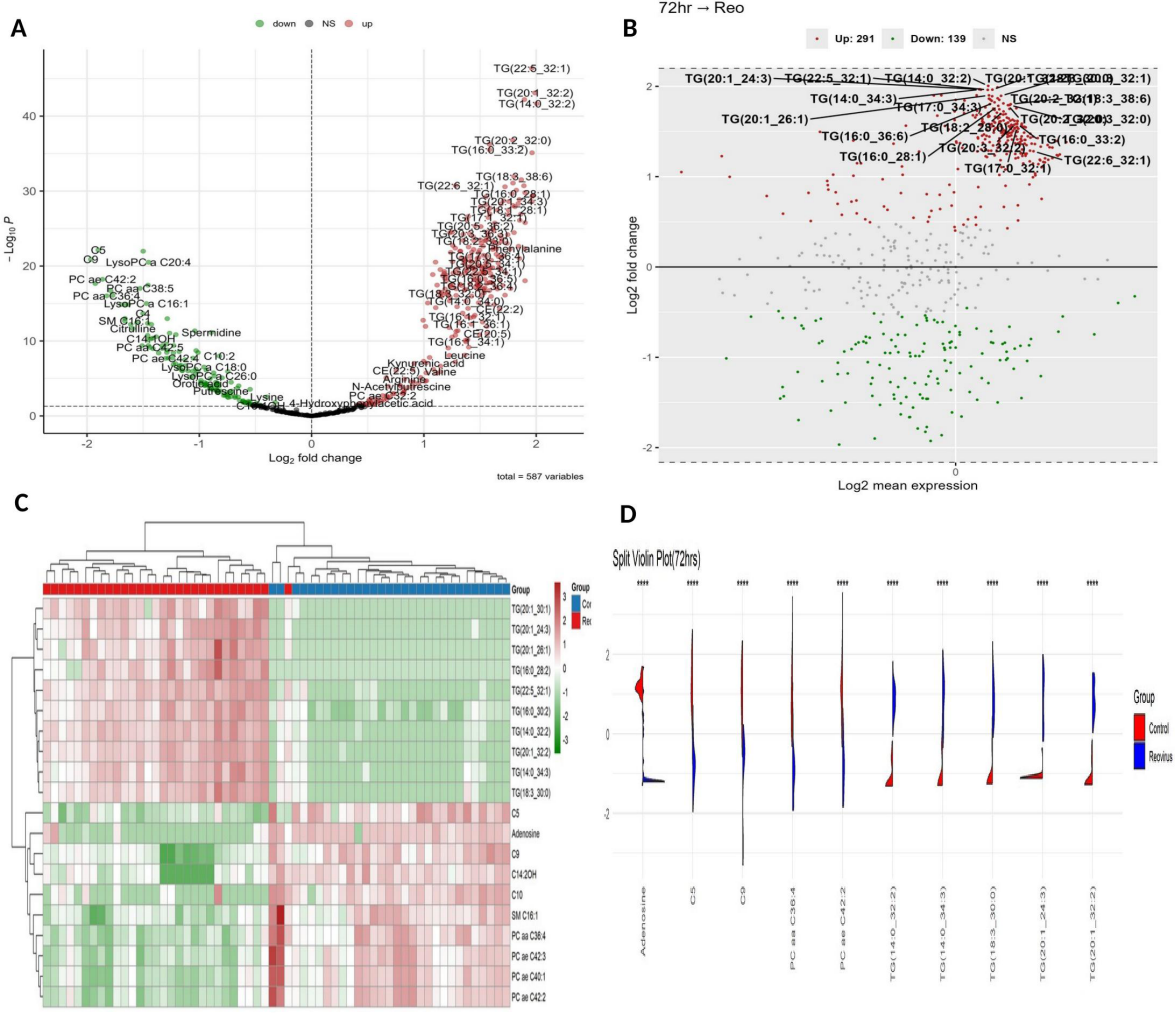
Metabolic alterations 72 h post ARV infection. **(A)** Volcano plot of 402 significantly altered metabolites (276 upregulated, 126 downregulated; *P*_adj_ < 0.05, FC > |1.5|). **(B)** MA plot highlighting the strong upregulation of triglycerides (e.g., TG 20:1_32:2, TG 14:0_34:3) and the suppression of nucleotides (adenosine, uridine). **(C)** Heatmap revealing distinct group separation with elevated neutral lipids (TGs, cholesteryl esters) and reduced nucleotide and membrane-associated metabolites. **(D)** Violin plots confirming lipid subclass enrichment (acylcarnitines, PCs, TGs) and the depletion of purine metabolites in infected samples.

At 72 h post-infection, a total of 402 differentially expressed metabolites (DEMs) were identified, of which 276 (68.65%) were upregulated and 126 (31.34%) were downregulated based on a statistical cutoff of *P*_adj_ < 0.05 and fold change >|1.5| (**Figure 5A**). Of the 402 metabolites, 79% were lipids—mainly TG, phosphatidylcholines, and cholesteryl esters. The non-lipids (21%) included amino acids, acylcarnitines, organic acids, and nucleotides. The mean fold change for the upregulated metabolites was 1.40 ± 0.30, while the downregulated metabolites had a mean fold change of -1.10 ± 0.34, indicating moderate variability in metabolic shifts. The distribution plots of log_2_ fold change (logFC) (**Supplementary Figure S3E**) and mean expression (**Supplementary Figure S3F**) further illustrate the overall metabolic alterations. The logFC distribution shows a broad spread with a clear shift toward upregulated metabolites, while the mean expression distribution displays a unimodal pattern with most metabolites clustered around low average expression values, with a longer tail toward a higher expression, which was consistent with prior timepoints.

The MA plot (**Figure 5B**) further visualized the log2 fold changes relative to the average expression levels, where upregulated lipids, particularly TG such as TG (20:1_32:2) and TG (14:0_34:3), clustered prominently at moderate-to-high abundance, whereas downregulated metabolites like adenosine and uridine appeared at a relatively lower mean expression. These plots confirmed that the metabolic response at 72 h was skewed toward strong lipid upregulation and nucleotide suppression. The heatmap analysis (**Figure 5C**) revealed a clear separation between the control and infected groups, with distinct clustering patterns driven by the upregulation of neutral lipids (e.g., TG, cholesteryl esters) and the downregulation of nucleotide-related and membrane-associated metabolites (e.g., LysoPC a C26:0, adenosine). Supporting this, the split-violin plots (**Figure 5D**) highlighted elevated distributions for lipid subclasses such as acylcarnitines (C5, C9), phosphatidylcholines (PC ae C42:2), and numerous TGs, while a consistent decline in features like adenosine and other purine derivatives reinforced the suppression of nucleotide metabolism. These observations together underscore a widespread lipid remodeling coupled with depletion of nucleotide pools, reflecting advanced viral manipulation of host energy and membrane biosynthesis pathways by 72 h post-infection.

### 3.4 Comparative analysis of metabolites at 24, 48, and 72 h post-ARV infection

**Figure 6:**
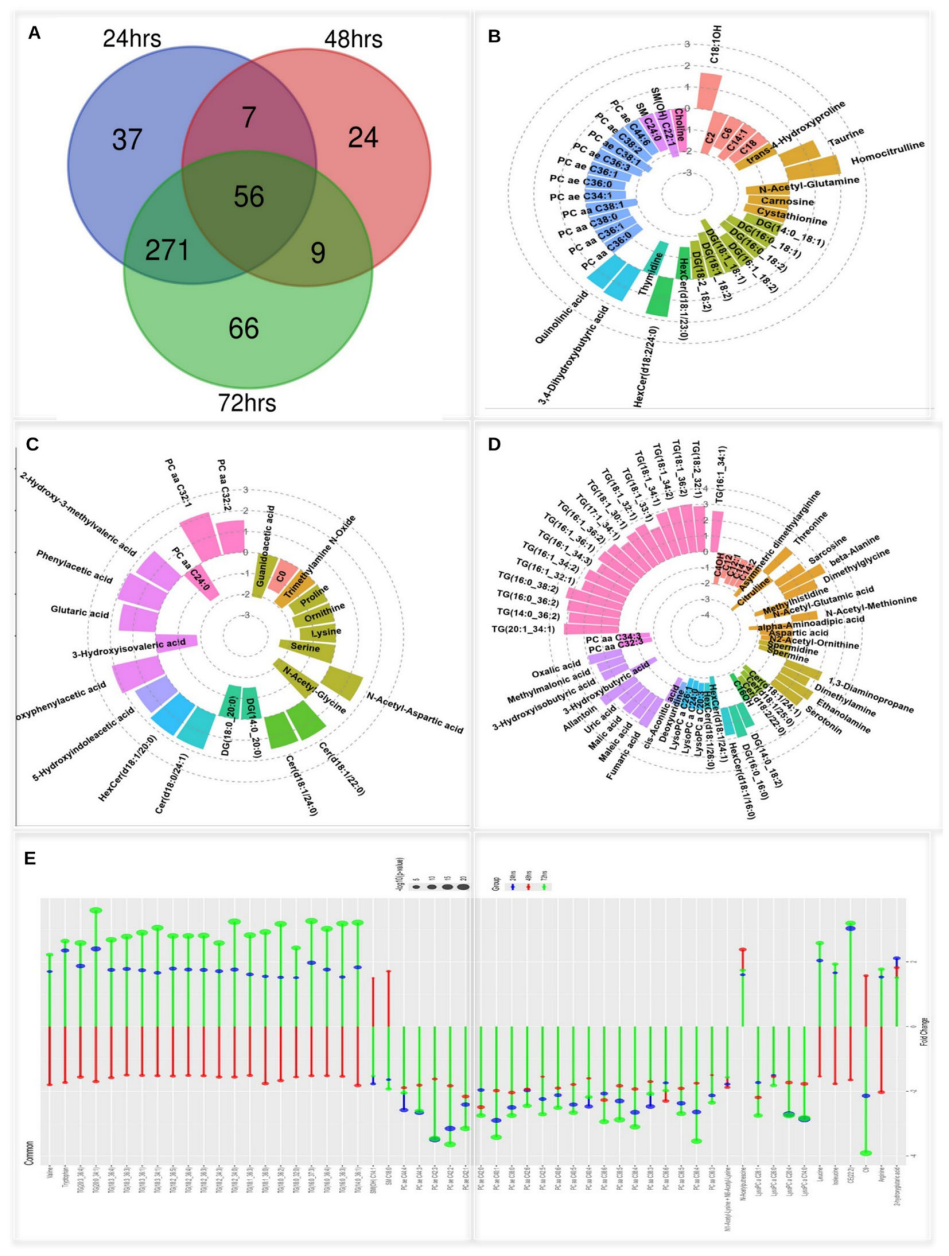
Comparative metabolite analysis across 24, 48, and 72 h post-ARV infection. **(A)** Venn diagram showing the unique and shared differentially expressed metabolites at each timepoint (37 at 24 h, 24 at 48 h, 66 at 72 h, and 56 common across all). **(B–D)** Circular plots of unique metabolites at 24, 48, and 72 h, illustrating the predominant lipid downregulation at 24 h, balanced regulation at 48 h, and strong triglyceride upregulation with amino acid and sphingolipid downregulation at 72 h. **(E)** Forest plot of 56 common metabolites, revealing the consistent suppression of phosphatidylcholines and LysoPCs, alongside the dynamic regulation of triglycerides, branched-chain amino acids, and stress-related metabolites.

ARV infection induces stage-specific metabolic shifts, with 37 unique metabolites altered at 24 h, 24 at 48 h, and 66 at 72 h, reflecting early, transitional, and late-phase responses (**Supplementary Table S1D**; **Figure 6A**).

At 24 h post-infection, 84% (31/37) of the unique metabolites were downregulated (e.g., DG (18:1_18:1), SM(OH) C22:1, PC ae C38:1; FC < –2.1), reflecting lipid and nucleotide suppression. Only 16% (6/37) were upregulated (e.g., 3,4-dihydroxybutyric acid, quinolinic acid, taurine; FC > 1.5), indicating early stress responses (**Figure 6B**). At 48 h post-infection, 50% (12/24) of the unique metabolites were upregulated (e.g., PC aa C32:1, 4-hydroxyphenylacetic acid; FC > 2.1), while 50% were downregulated (e.g., serine, N-acetyl-glycine; FC < –2.1), reflecting a transitional phase marked by amino acid suppression and compensatory lipid and organic acid responses (**Figure 6C**). At 72 h post-infection, 57.6% (38/66) of the unique metabolites were upregulated (e.g., TG(16:0_38:2), TG(20:1_34:1), TG(16:0_36:2); FC > 3.0), indicating intensified lipid accumulation, while 42.4% (28/66) were downregulated (e.g., HexCer (d18:1/24:1), asymmetric dimethylarginine, citrulline; FC < –2.0), reflecting suppressed amino acid and sphingolipid metabolism (**Figure 6D**; **Supplementary Table S1E**).

A core set of 56 metabolites was consistently dysregulated across all timepoints, defining a robust metabolic signature of ARV infection. This shared signature persisted despite key experimental differences between the two challenge experiments, including ARV genotypic cluster (CII vs. CVI), host age at infection (7 vs. 12 days post-hatch), and post-infection sampling timepoints (24 and 72 hpi vs. 48 hpi), underscoring the reproducibility and reliability of this core ARV-induced metabolic response. Additionally, seven and nine metabolites were shared between 24–48 and 48–72 h, respectively, while a large overlap (*n* = 271 metabolites) was observed between 24 and 72 h, suggesting that early metabolic alterations are sustained or intensify as the infection progresses.

An analysis of the 56 common metabolites revealed both consistent and time-dependent metabolic alterations across reovirus infection. TG exhibited a biphasic regulation pattern, showing modest upregulation at 24 h and a relative decrease or suppression at 48 h, followed by a pronounced and robust surge at 72 h, reflecting dynamic shifts in lipid storage and energy metabolism. Conversely, phosphatidylcholines (PC aa and PC ae) and LysoPC were uniformly downregulated at all timepoints, with the magnitude of suppression progressively deepening from 24 to 72 h, which was indicative of sustained perturbation in membrane lipid homeostasis (**Figure 6E**). The branched-chain amino acids, notably leucine and isoleucine, along with cholesteryl esters, demonstrated a characteristic temporal fluctuation, increasing at 24 and 72 h but transiently diminishing at 48 h, suggesting a metabolic inflection during the mid-infection phase. Markers of oxidative and polyamine stress, including 2-hydroxyglutaric acid and N-acetylputrescine, were elevated throughout the infection course, attaining peak abundance at 72 h, while the N1-/N6-acetyl-lysine levels remained consistently suppressed, reflecting ongoing modifications in protein acetylation dynamics.

These findings together establish a dynamic yet coherent metabolic trajectory of ARV infection, anchored by phospholipid depletion, escalating oxidative stress, and time-specific modulation of lipid and amino acid metabolism.

### 3.5 Pathway analysis of metabolites

Pathway enrichment analysis at 24, 48, and 72 h post-infection revealed statistically and biologically significant shifts in host metabolism based on statistical (p-values) and pathway impact scores (topological relevance).

**Figure 7:**
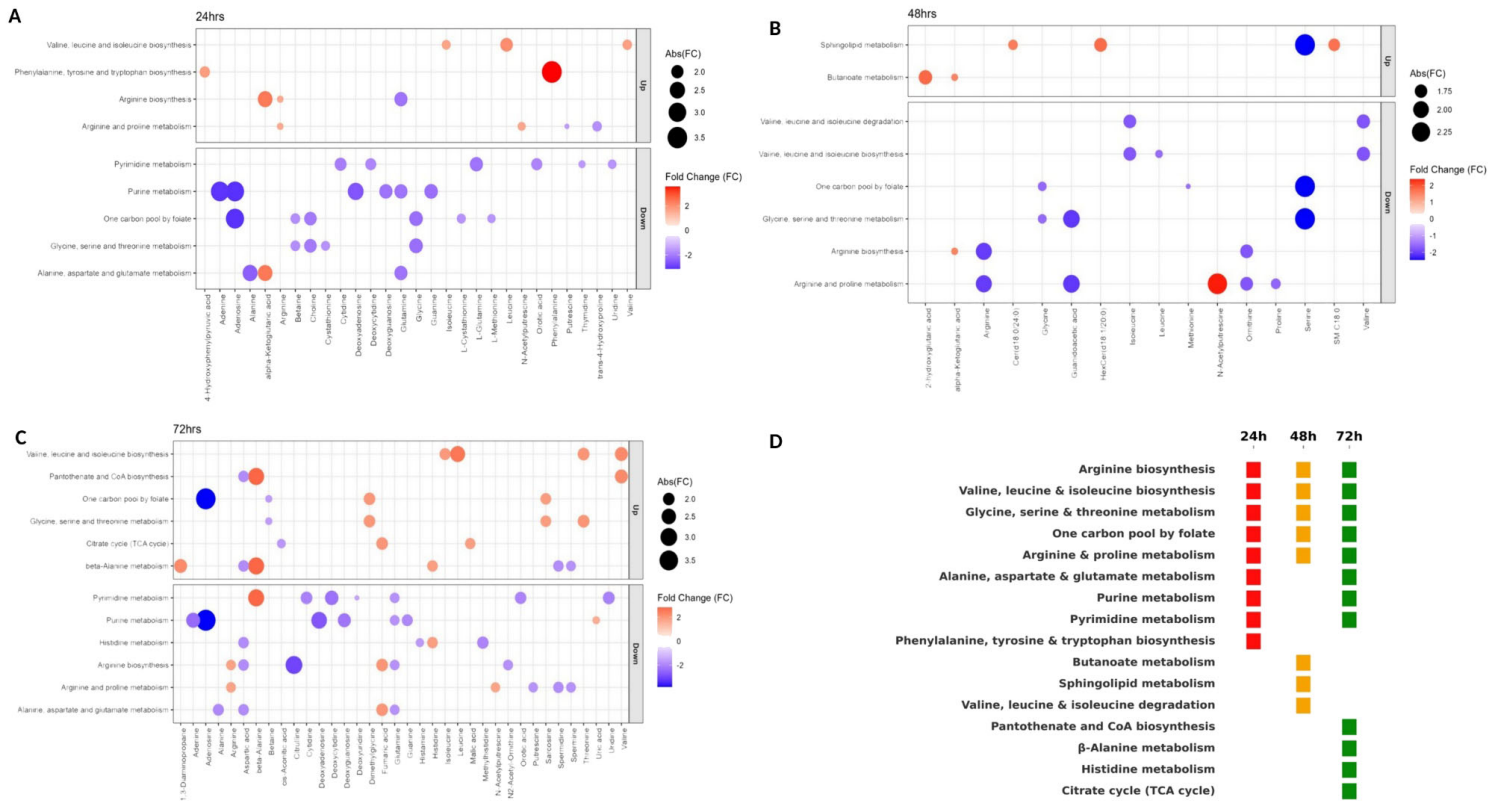
Pathway enrichment analysis of metabolites at 24, 48, and 72 h post-ARV infection. **(A)** At 24 h, significant downregulation of nucleotide-related pathways (e.g., one carbon pool by folate, pyrimidine metabolism) occurs alongside the upregulation of amino acid biosynthesis (valine, leucine, isoleucine; phenylalanine, tyrosine, tryptophan). **(B)** At 48 h, the upregulation of sphingolipid and butanoate metabolism contrasts with the stronger suppression of amino acid pathways, including valine/leucine/isoleucine biosynthesis and arginine/proline metabolism. **(C)** At 72 h, broad metabolic rewiring is observed with upregulated β-alanine metabolism, TCA cycle, amino acid biosynthesis, and one-carbon metabolism, alongside the suppression of arginine, pyrimidine, and histidine metabolism. The bubble size indicates pathway impact; the color reflects direction and fold change magnitude. **(D)** Summary heatmap showing significantly altered pathways across 24, 48, and 72 h post-infection, highlighting the temporal shift from early pathway suppression to later metabolic activation.

At 24 h post-infection, the enrichment analysis revealed early metabolic reprogramming (**Supplementary Table S2A**; **Figure 7A**). The most significant downregulation occurred in nucleotide-related pathways, including the one carbon pool by folate (6/26 metabolites, *p* = 6.08 × 10^-5^, impact = 0.22) and pyrimidine metabolism (6/40, *p* = 7.48 × 10^-4^, impact = 0.14), suggesting reduced methylation potential and impaired nucleotide synthesis. In contrast, the amino acid biosynthetic pathways were prominently upregulated, including valine, leucine, and isoleucine biosynthesis (3/8, *p* = 1.14 × 10^-^³, impact = 0.00) and phenylalanine, tyrosine, and tryptophan biosynthesis (2/4, *p* = 4.71 × 10^-^³, impact = 0.50). These alterations highlight an early host–virus interface where reovirus suppresses nucleotide metabolism while simultaneously driving amino acid biosynthesis to favor viral replication and cellular adaptation.

By 48 h post-infection, the metabolic profile indicated further disruption. Here sphingolipid metabolism (4/32, *p* = 0.0025, impact = 0.347) and butanoate metabolism (2/15, *p* = 0.0308, impact = 0) were upregulated, suggesting a shift toward lipid signaling and short-chain fatty acid metabolism. In contrast, downregulation became more pronounced in amino-acid-related pathways: valine, leucine, and isoleucine biosynthesis (3/8, *p* = 0.0003, impact = 0), arginine and proline metabolism (5/36, *p* = 0.0004, impact = 0.399), and one-carbon pool by folate (3/26, *p* = 0.0116, impact = 0.112), reflecting growing host biosynthetic suppression (**Supplementary Table S2B**; **Figure 7B**).

At 72 h post-infection, pathway enrichment showed extensive and coordinated metabolic rewiring. The upregulated pathways included β-alanine metabolism (6/21, *p* = 6.82 × 10^-5^, impact = 0.455), valine, leucine, and isoleucine biosynthesis (4/8, *p* = 1.05 × 10^-4^, impact = 0), one carbon pool by folate (4/26, *p* = 0.0136, impact = 0.212), and the citrate cycle (TCA cycle) (3/20, *p* = 0.0348, impact = 0.124). These points to heightened amino acid biosynthesis, methylation activity, and energy metabolism, supporting viral persistence. Meanwhile, the downregulated pathways included arginine biosynthesis (6/13, *p* = 2.72 × 10^-6^, impact = 0.354), arginine and proline metabolism (5/36, *p* = 0.0090, impact = 0.424), pyrimidine metabolism (7/40, *p* = 4.61 × 10^-4^, impact = 0.192), and histidine metabolism (4/16, *p* = 0.00218, impact = 0.410), suggesting the persistent suppression of nitrogen disposal and nucleotide turnover (**Supplementary Table S2C**; **Figure 7C**).

Across all of the infection stages, five pathways were consistently enriched throughout the infection course: arginine biosynthesis, valine/leucine/isoleucine biosynthesis, glycine/serine/threonine metabolism, one-carbon pool by folate, and arginine and proline metabolism (**Supplementary Table S2D**; **Figure 7D**). Their persistence across the early, intermediate, and late stages highlights a core metabolic signature of reovirus infection, reflecting sustained perturbations in amino acid and one-carbon metabolism that underpin viral replication and host stress responses.

### 3.6 Identification of ARV metabolite biomarkers

To identify shared and predictive biomarkers of ARV infection, LR was applied to 56 differentially expressed metabolites identified from the analysis of 24-, 48-, and 72-h post-infection data. The resulting effect size (log-odds coefficient) indicates association strength and direction; positive values represent infection-associated metabolites, while negative values indicate protective depletion.

#### 3.6.1 Persistent biomarkers

In the case of the shared 56 metabolites, the LR results show that two metabolites were consistently upregulated at all timepoints (**Supplementary Table S2E**). N-Acetylputrescine showed strong positive associations with infection, with log-odds estimates of +1.64 (*p* = 0.000843) at 24 h, +1.38 (*p* = 0.000695) at 48 h, and +1.29 (*p* = 0.000770) at 72 h, corresponding to odds ratios (OR) of ~5.1, 4.0, and 3.6, respectively. These values indicate that each 1-µM increase in N-acetylputrescine concentration increased the probability of infection classification by more than threefold across all stages. Similarly, 2-hydroxyglutaric acid remained significantly elevated, with effect sizes of +1.37 (*p* = 0.000750), +1.20 (*p* = 0.001879), and +1.15 (*p* = 0.006288) at 24, 48, and 72 h, respectively, corresponding to ORs ranging from 3.9 to 3.1 per micromolar increase. In contrast, a broad set of phospholipids exhibited persistent depletion across all stages (**Supplementary Figures S4A, C, E**). Notably, LysoPC a C14:0 was strongly downregulated at 24 h (−8.89, *p* = 0.0062), 48 h (−3.23, *p* = 0.00070), and 72 h (−7.80, *p* = 0.0027). LysoPC a C20:4 followed a similar pattern with estimates of −9.67 (*p* = 0.0090), −3.74 (*p* = 0.00070), and −5.66 (*p* = 0.00090) across the respective timepoints. PC aa C38:5 was likewise consistently depleted (−6.17, *p* = 0.0023; −4.20, *p* = 0.0010; −4.12, *p* = 0.00036) as was PC ae C42:3 (−4.29, *p* = 0.00075; −1.85, *p* = 0.0026; −4.25, *p* = 0.00085). Collectively, these findings demonstrate that ARV infection induces the sustained elevation of polyamine (N-acetylputrescine) and stress-related metabolites (2-hydroxyglutaric acid) while simultaneously depleting lysophosphatidylcholines and phosphatidylcholines that are critical for membrane integrity and immune signaling (**Supplementary Figures S4A, C, E**).

#### 3.6.2 Stage-specific biomarkers

LR identified unique stage-specific biomarkers of ARV infection. At 24 h, the top positive predictor was homocitrulline (log-odds = +5.70, *p* = 0.0009; OR ≈ 299), and the strongest negative predictor was DG (18:1_18:1) (−4.08, *p* = 0.00094; OR ≈ 0.017) (**Supplementary Table S2F**; **Supplementary Figures S4B, D, F**). At 48 h, lysine emerged as the dominant negative biomarker (−3.42, *p* = 0.0013; OR ≈ 0.033), whereas 4-hydroxyphenylacetic acid was the strongest positive predictor (+1.92, *p* = 0.0013; OR ≈ 6.85) (**Supplementary Table S2G**). By 72 h, lipid accumulation was evident, with TG (18:1_33:1) showing the most pronounced increase (log-odds = +8.21, *p* = 0.0025; OR ≈ 3665), while citrulline was the leading depleted metabolite (−3.92, *p* = 0.00084; OR ≈ 0.020) (**Supplementary Table S2H**; **Supplementary Figures S4B, D, F**). Collectively, these stage-specific biomarkers reveal a temporal trajectory in ARV infection, early oxidative and lipid perturbations at 24 h, amino acid depletion with stress metabolite elevation at 48 h, and severe triglyceride accumulation with immune suppression at 72 h.

#### 3.6.3 Biomarker performance analysis

**Figure 8:**
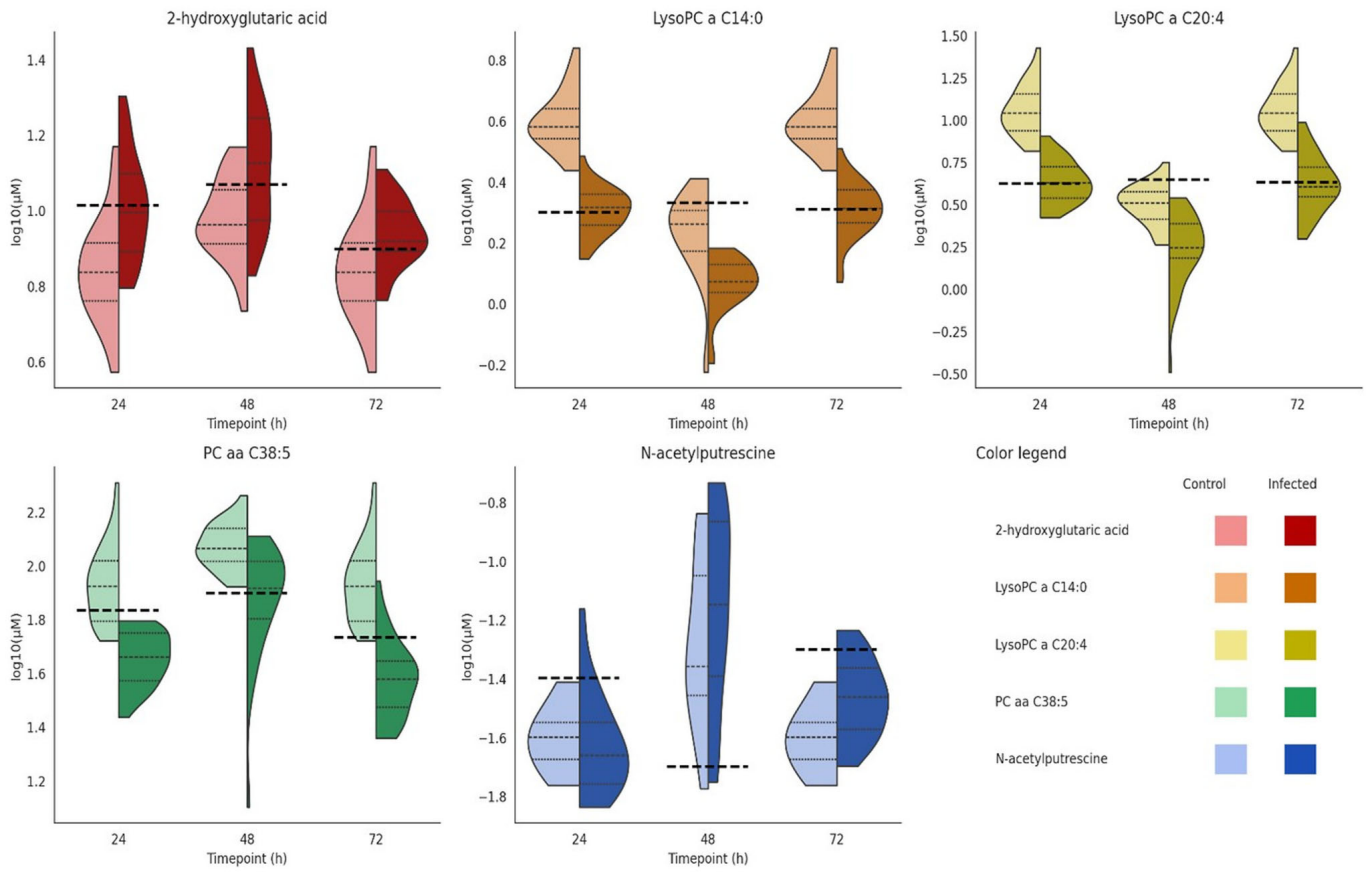
Split violin plots of metabolite biomarker concentrations with timepoint-specific cutoffs. Each panel shows the log10(µM) concentrations of metabolite concentrations at 24, 48, and 72 h for the control and infected chicken samples. For each timepoint and group, three solid horizontal lines display the 25th percentile (Q1), median, and 75th percentile (Q3) of the respective distribution. The overlaid dashed line indicates the timepoint-specific diagnostic cutoff. The width of each violin reflects the local density of the data, and individual sample measurements are shown as jittered points.

Following logistic-regression screening, five biomarkers showed a robust discrimination between the infected and control sera at 24, 48, and 72 h. LysoPC a C14:0, LysoPC a C20:4, and PC aa C38:5 each achieved AUC ≈ 1.00, with high sensitivity/specificity at their Youden-optimal micromolar cutoffs (e.g., LysoPC a C14:0 sensitivity 0.93–0.97, specificity 0.83–1.00, cutoff ~2.00–2.14 μM). N-Acetylputrescine also performed strongly (AUC 0.965–1.00) with sensitivity up to 1.00, albeit with more modest specificity (~0.67–0.83) at low micromolar cutoffs (~0.02–0.05 μM), consistent with its infection-associated increase. 2-Hydroxyglutaric acid had AUC 0.999–1.00 but with a wider variation in sensitivity (~0.47–0.90) and specificity (~0.57–0.97) across timepoints at cutoffs near 8–12 μM (**Supplementary Table S2I**). Split violins of log10(µM) show time- and biomarker-specific differences between the control and ARV-infected groups. Quartile lines summarize the central tendency and spread, and dashed lines mark timepoint-specific cutoffs. Several biomarkers show clear group separation at one or more timepoints, while others overlap, illustrating heterogeneity across markers and time (**Figure 8**).

## 4 Discussion

ARV infection poses a significant threat to poultry health and economy, causing viral arthritis that leads to lameness and production losses (Sellers, 2022). Early detection is difficult with traditional methods, but serum metabolomics can identify host–pathogen interactions before the onset of clinical signs. Our previous study showed that targeted metabolomics can identify infection biomarkers within 24 h, enabling the early detection of *Escherichia coli* sepsis before culture results are available (Ranaraja et al., 2025). Our findings demonstrate that ARV infection induces persistent and stage-specific metabolic changes that parallel tissue damage and clinical signs in chickens.

Arthrotropic ARV primarily affects the tendons, fibrocartilage, and the synovial membrane of broiler chickens, causing tenosynovitis/arthritis. Following infection, the virus replicates in the fibroblasts, macrophages, and the synoviocytes of tendon sheaths, leading to CPEs like cell lysis and syncytium formation. Viral replication activates the innate immune system first, causing the release of proinflammatory cytokines [interferon-γ (IFN-γ), interleukin-1β (IL-1β), and tumor necrosis factor-α (TNF-α)] by dendritic cells and macrophages, which further attract heterophils and mononuclear cells to infiltrate into the tendon sheath. Later, adaptive immune cells, mainly clusters of differentiation (CD)8+ T cells, move into the infected tendons (Chen et al., 2014; Nour and Mohanty, 2024; Hsueh et al., 2025). Our previous study, by Ayalew et al., demonstrated that lesion severity and CD8+ T cell infiltration correlated with the virulence of the infected ARV (Ayalew et al., 2022). Overall, these reactions contribute to the inflammatory response and the tissue damage at the infection site, leading to swollen foot pads, lameness, and, sometimes, rupture of the gastrocnemius tendons.

Our LR modeling has identified five serum metabolite signatures comprising lipids (LysoPC a C14:0, LysoPC a C20:4, and PC aa C38:5) and amines (N-acetylputrescine-NAP) and organic acids (2-hydroxyglutaric acid, 2-HG), which were consistently altered across all timepoints. The three lipid metabolites, LysoPC a C14:0, LysoPC a C20:4, and PC aa C38:5, showed a consistent decline at 24, 48, and 72 h after ARV challenge and functioned as clear diagnostic readouts. Each operated at low-micromolar decision thresholds (C14:0 ~2.0–2.14 μM, C20:4 ~4.20–4.43 μM, and PC aa C38:5 ~54–79 μM) and achieved strong single-analyte discrimination across time, with near-perfect rule-in specificity at 24 and 72 h and preserved performance at 48 h. This behavior is biologically expected, as during infection and systemic inflammation the circulating lysophosphatidylcholines and phosphatidylcholines commonly fall, which might be due to membrane turnover, immune–lipid crosstalk, and altered lipoprotein trafficking (Dai et al., 2024; Liu et al., 2024; Wang et al., 2024; Ma et al., 2022).

In ARV specifically, the p10 FAST protein induces cell–cell fusion and membrane injury, plausibly accelerating phospholipid consumption and lowering the serum LysoPC/PC pools (Ciechonska and Duncan, 2014; Salsman et al., 2005; Kanai et al., 2019). More broadly, manipulation of host membrane lipids to facilitate viral replication, assembly, and cell-to-cell spread is a common strategy among many viruses, suggesting that the LysoPC/PC depletion observed here may reflect a broader virus-driven membrane remodeling response. A recent human study involving severe fever with thrombocytopenia syndrome (SFTS) caused by bunyavirus revealed a significant alteration of the patient’s lipid profiles compared to healthy controls (Guo et al., 2024). According to this study, the LysoPC levels of SFTS patients showed a consistent downregulation from mild infection to critical stages. At the same time, they discussed a negative correlation between IL-6-like inflammatory mediators and the LysoPC levels, revealing that LysoPC reduction could be related to the inflammatory status of the body. This closely represents the findings of our study, where LysoPC was consistently downregulated from mild to severe infection, going from 24 to 72 h. Complementary cell work in chickens also shows virus-driven remodeling of membrane phospholipids and host PC synthesis pathways, supporting the mechanistic link from viral replication and fusion to phospholipid loss (Gimenez et al., 2021). This study, done in quail myoblast-derived fibroblast-like cell line, demonstrated that the avian infectious bursal disease viral protein 3 (VP3) localizes to PI3-phosphate-enriched early endosomes to form replication complexes. These findings further support our study that chicken RNA viruses hijack host membrane lipids for their replication, hence the possible downregulation (Gimenez et al., 2021).

Furthermore, LysoPC is known to destabilize lipid membranes by introducing a positive curvature into the lipid bilayer due to its inverted cone shape. At high concentrations, it can impair the fusion of viruses like tick-borne encephalitis with host membranes (Stiasny and Heinz, 2004). Hence, the depleted LysoPc favors viral spread by stabilizing the membranes. In addition, a study done using a human embryonic kidney cell line found that LysoPC can induce innate immunity by binding with toll-like receptors (TLR2/4) and produce proinflammatory cytokines such as IL-8 through the NF-κB pathway (Carneiro et al., 2013). Therefore, the depletion of LysoPC may reflect ARV-driven membrane destabilization, heightened inflammatory signaling, and oxidative stress, collectively contributing to the pathological lesions observed following the ARV challenge. Nevertheless, these mechanistic links remain hypothetical in the context of the present study and require direct experimental validation.

In this study, we identified that NAP was consistently upregulated across all timepoints (24, 48, and 72 h post-infection). In our data, NAP showed high discrimination (AUC ~0.97–1.00) with simple, low-micromolar decision thresholds: >0.019 to >0.046 µM. However, its sensitivity and specificity were modest (~0.67–0.83), making NAP a rule-out analyte that reduces missed cases and ideally paired with a high-specificity lipid for balanced decisions. NAP, the acetylated breakdown product of putrescine, indicates the activation of the spermidine/spermidine N1-acetyltransferase (SAT1)-driven polyamine catabolism pathway, which is stimulated by interferon to restrict viral replication in RNA viruses like Chikungunya and Zika virus in human and mouse-derived cell lines (Mounce et al., 2016, 2017). Sustained levels of NAP from 24–72 h post-infection align with IFN-γ-rich tendon inflammation seen in ARV tenosynovitis. This supports the existing evidence that activating the SAT1-driven polyamine pathway limits RNA virus growth (Cruz-Pulido et al., 2023; Ayalew et al., 2022).

On the other hand, viruses, including ARV, depend on host polyamines for replication and protein translation, while the host utilizes polyamines for immune responses, leading to enhanced polyamine catabolism in the body. Therefore, an increase in NAP might suggest rapid polyamine turnover for viral replication and host immune responses. Moreover, polyamine catabolism via the SAT1 pathway generates reactive intermediates such as hydrogen peroxide. Thus, the increment in NAP can also be related to enhanced viral replication and oxidative tissue damage during the infection (Firpo and Mounce, 2020; Stewart et al., 2018).

The second non-lipid metabolite, 2-HG, a by-product of TCA-cycle/redox imbalance, also remained elevated at all three timepoints. 2-HG had high AUC but time-variable sensitivity/specificity at ~8–12 μM; we therefore use it as a supportive (rule-out) marker alongside a high-specificity lipid to boost the overall performance. This finding could be consistent with hypoxia/pseudohypoxia-driven L-2HG production and mitochondrial stress (Intlekofer et al., 2015; Oldham et al., 2015). Its persistence is compatible with leukocyte activation and tissue inflammation, as L-2HG can activate HIF-1α and pro-inflammatory, glycolytic programs in macrophages, while D-2HG accumulates after TLR4 activation (Williams et al., 2022; de Goede et al., 2022). The rise we observed also fits the ARV effects in chicken fibroblasts, where proteomics shows stress response and energy metabolism reprogramming during infection; together with the inflammatory tendon lesions typical of ARV tenosynovitis, this provides a coherent mechanistic backdrop for 2-HG elevation (Chen et al., 2014; Sellers, 2022). Taken together, all five operate at low-micromolar concentrations with simple directions of change (lipids ↓, NAP/2-HG ↑), supporting the field translation on routine LC–MS/MS. Taken together, these persistent signals summarize the metabolic features of ARV pathology in chickens, polyamine turnover indicative of antiviral immunity, oxidative stress/pseudohypoxia marked by 2-HG, and phospholipid depletion that likely contributes to tendon injury and clinical lameness, providing a succinct, clinically usable basis for screening and confirmation across 24 to 72 h.

Our pathway analysis identified five metabolic routes consistently altered in ARV-infected chickens: arginine metabolism, arginine and proline metabolism, branched-chain amino-acid (valine, leucine, and isoleucine) pathway activity, glycine/serine/threonine metabolism, and the one-carbon (folate) pool. Reduced arginine availability would be expected to lower nitric oxide production by myeloid cells, weakening antimicrobial activity and tissue repair and potentially contributing to lesion formation and delayed healing (Rodríguez et al., 2017). Shifts in arginine↔proline directly affect collagen synthesis and extracellular matrix integrity, consistent with tendon and synovial damage and lameness in ARV infection (Albaugh et al., 2017). Altered BCAA pathway activity reflects changed immune-cell energy and anabolic demands, as BCAAs support lymphocyte proliferation and function during infection (Calder, 2006). Changes in glycine/serine/threonine metabolism can limit the nucleotide supply and redox balance, which are critical for rapidly dividing and activating immune cells under inflammatory stress (Ma et al., 2017). Finally, disturbances in the one-carbon/folate pathway align with viral strategies that hijack the host methyl-donor and nucleotide biosynthesis for replication and host-gene control (Zhang et al., 2021). These findings together outline how ARV disrupts host metabolism in ways that impair immune defense, tissue integrity, and cellular functions needed to resolve an infection. These pathway-level interpretations provide biological context for the observed metabolite changes, although they should be viewed as inference-based rather than direct functional proof.

The strengths of this study include its temporal design (24, 48, and 72 h), the use of LR modeling to identify predictive metabolites, and the integration with pathway analysis. These provide robust biomarker candidates for early diagnosis. Limitations include a lack of tissue-level validation, the absence of *in vitro* experiments, and study conditions that may not fully reflect field variability. Although the sustained depletion of LysoPC/PC species points toward ARV-driven membrane remodeling, this remains associative rather than a mechanistically validated one. Furthermore, the diagnostic specificity of the proposed biomarkers against other poultry pathogens with overlapping clinical signs remains to be established. In addition, although the metabolomic changes paralleled tissue damage and clinical signs, a direct correlation analysis between serum metabolite levels and histopathological lesion severity was not performed. Future studies should investigate the relationship between serum markers and tendon pathology, test whether modulating arginine–polyamine or lipid metabolism alters disease outcomes, and carry out validation of the potential biomarkers in field conditions.

In conclusion, this study demonstrated that serum metabolomics is an effective experimental approach to detect and track the early and ongoing changes to the metabolic reprogramming caused by ARV infections in chickens. Our data have shown consistent changes due to ARV infection over time (from 24 through 72 h post-infection) using a stable group of biomarkers. The lipids (LysoPC a C14:0, LysoPC a C20:4, and PC aa C38:5) decreased at all three points in time, establishing them as excellent candidate biomarkers to identify ARV infection. In addition, increases in N-acetylputrescine and 2-hydroxyglutarate identified by our serum metabolomics analysis indicated elevated levels of polyamine catabolism and redox/mitochondrial stress, respectively. These findings together identify promising candidate biomarkers for the early detection of ARV-associated viral arthritis and provide new insight into the host metabolic alterations associated with an infection. Metabolomic surveillance is a viable method to enhance the diagnostic monitoring of ARV and future metabolism-informed interventions.

## Data Availability

The original contributions presented in the study are included in the article/**Supplementary Material**. Further inquiries can be directed to the corresponding author.

## Supplementary material

Supplementary Figure S1 Boxplots of metabolomics data before and after normalization. **(A)** Raw intensity values across all samples displayed high variability and skewed distributions, reflecting technical and experimental noise. **(B)** Following normalization (median scaling, log_2_ transformation, and z-standardization), the data showed consistent scaling across groups, with distributions centered around zero and reduced variability. These preprocessing steps improved the comparability and reliability for downstream analyses.

Supplementary Figure S2 Principal component analysis (PCA) of metabolomics data before and after batch correction. **(A)** The PCA of the uncorrected dataset showed clustering primarily driven by batch effects, with PC1 (40.6% variance) and PC2 (12.4% variance) reflecting experimental batch rather than biological condition. **(B)** After batch correction using the sva package, batch-related variation was effectively minimized. PC1 (27.7% variance) and PC2 (16.4% variance) captured biologically relevant separation, with control and reovirus-infected groups clustering according to infection status, particularly at 72 h post-infection. This demonstrates that the correction successfully reduced the batch effects while preserving true biological variation.

Supplementary Figure S3 Distribution of log_2_ fold change and mean expression of significantly altered metabolites across ARV infection timepoints. **(A)** Density histogram of log_2_ fold change for significantly altered metabolites at 24 h post-infection. **(B)** Density histogram of mean expression for significantly altered metabolites at 24 h post-infection. **(C)** Density histogram of log_2_ fold change for significantly altered metabolites at 48 h post-infection. **(D)** Density histogram of mean expression for significantly altered metabolites at 48 h post-infection. **(E)** Density histogram of log_2_ fold change for significantly altered metabolites at 72 h post-infection. **(F)** Density histogram of mean expression for significantly altered metabolites at 72 h post-infection.

Supplementary Figure S4 Logistic-regression-based metabolite biomarkers of ARV infection. **(A)** Forest plot (24 h) showing the persistent upregulation of N-acetylputrescine and 2-hydroxyglutaric acid, with strong depletion of LysoPCs and PCs. **(B)** Forest plot (24 h) highlighting the stage-specific markers: homocitrulline (positive) and DG(18:1_18:1) (negative). **(C)** Forest plot (48 h) confirming the sustained elevation of N-acetylputrescine and 2-hydroxyglutaric acid with continued LysoPC/PC depletion. **(D)** Forest plot (48 h) showing lysine as the dominant negative marker and 4-hydroxyphenylacetic acid as the top positive predictor. **(E)** Forest plot (72 h) demonstrating persistent lipid remodeling with elevated N-acetylputrescine and 2-hydroxyglutaric acid and broad LysoPC/PC suppression. **(F)** Forest plot (72 h) highlighting stage-specific markers: strong TG(18:1_33:1) upregulation and citrulline depletion, consistent with impaired nitric oxide synthesis.
