# Supplementary figures and images for: Serum metabolomic profiling reveals LysoPC/PC depletion as a potential biomarker to detect avian reoviral infection in neonatal broiler chickens
Source: Front Cell Infect Microbiol. 2026 May 8;16:1750590. doi: 10.3389/fcimb.2026.1750590 (PMC13194372; doi:10.3389/fcimb.2026.1750590)

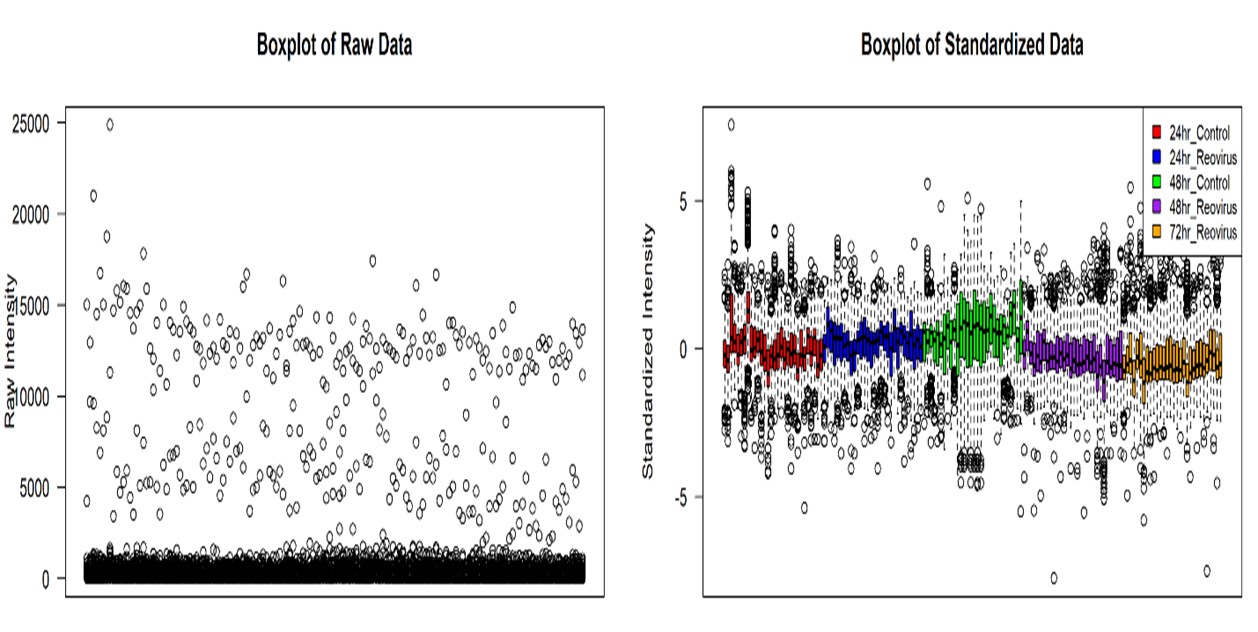

Supplement: Supplementary Figure S1 — Boxplots of metabolomics data before and after normalization. (A) Raw intensity values across all samples displayed high variability and skewed distributions, reflecting technical and experimental noise. (B) Following normalization (median scaling, log2 transformation, and z-standardization), the data showed consistent scaling across groups, with distributions centered around zero and reduced variability. These preprocessing steps improved the comparability and reliability for downstream analyses. [file Image1.jpeg]

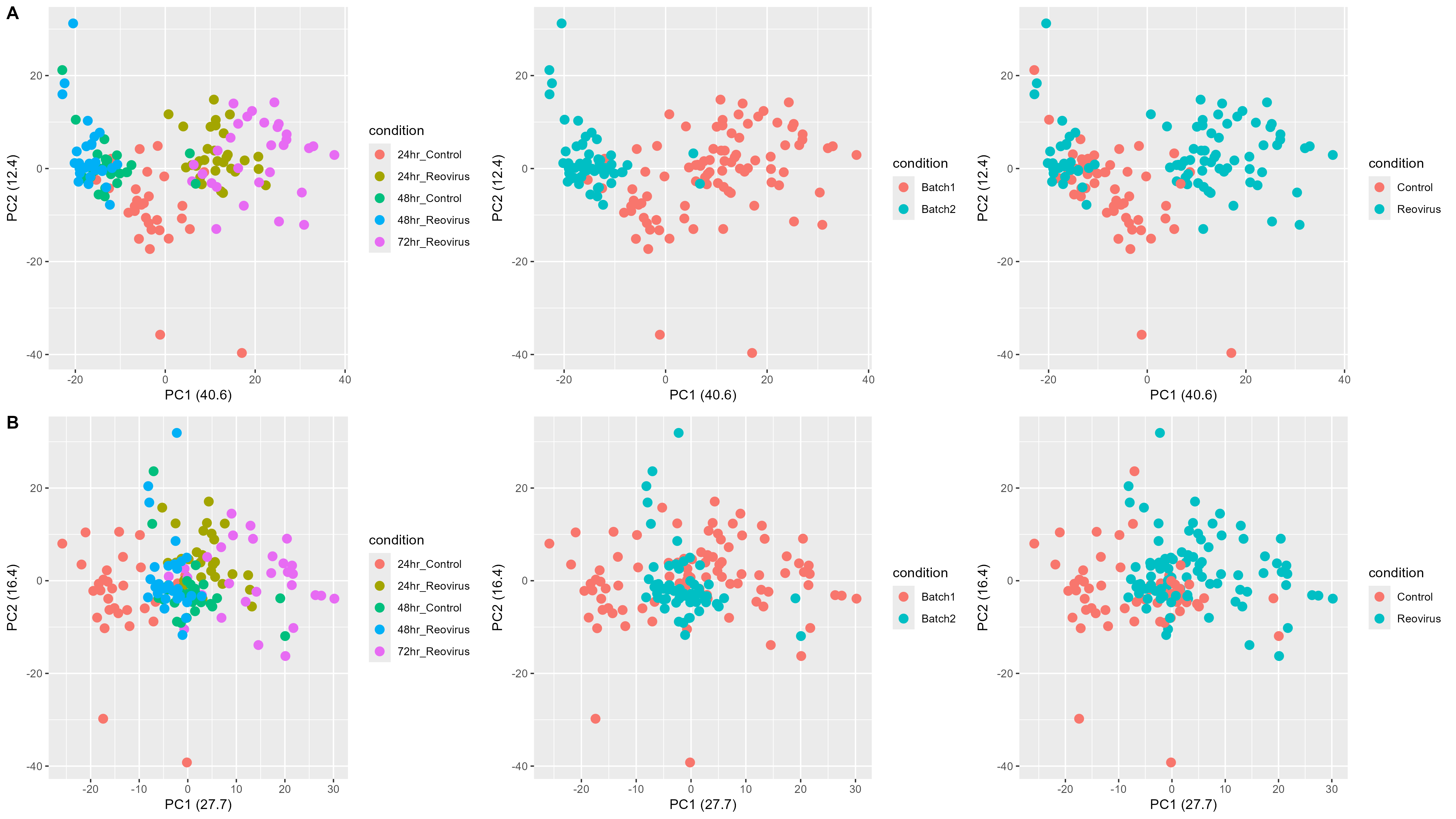

Supplement: Supplementary Figure S2 — Principal component analysis (PCA) of metabolomics data before and after batch correction. (A) The PCA of the uncorrected dataset showed clustering primarily driven by batch effects, with PC1 (40.6% variance) and PC2 (12.4% variance) reflecting experimental batch rather than biological condition. (B) After batch correction using the sva package, batch-related variation was effectively minimized. PC1 (27.7% variance) and PC2 (16.4% variance) captured biologically relevant separation, with control and reovirus-infected groups clustering according to infection status, particularly at 72 h post-infection. This demonstrates that the correction successfully reduced the batch effects while preserving true biological variation. [file Image2.png]

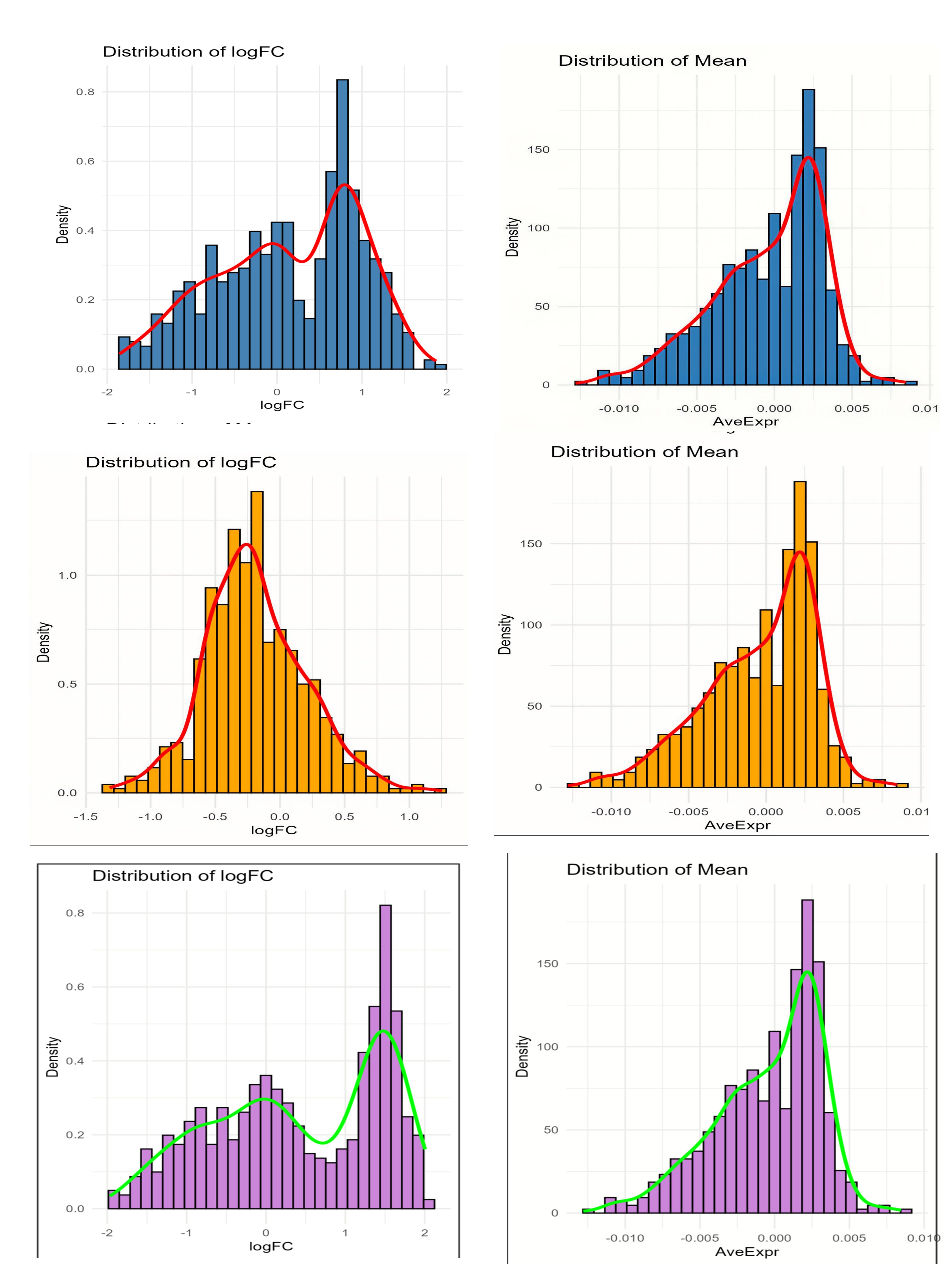

Supplement: Supplementary Figure S3 — Distribution of log2 fold change and mean expression of significantly altered metabolites across ARV infection timepoints. (A) Density histogram of log2 fold change for significantly altered metabolites at 24 h post-infection. (B) Density histogram of mean expression for significantly altered metabolites at 24 h post-infection. (C) Density histogram of log2 fold change for significantly altered metabolites at 48 h post-infection. (D) Density histogram of mean expression for significantly altered metabolites at 48 h post-infection. (E) Density histogram of log2 fold change for significantly altered metabolites at 72 h post-infection. (F) Density histogram of mean expression for significantly altered metabolites at 72 h post-infection. [file Image3.jpeg]

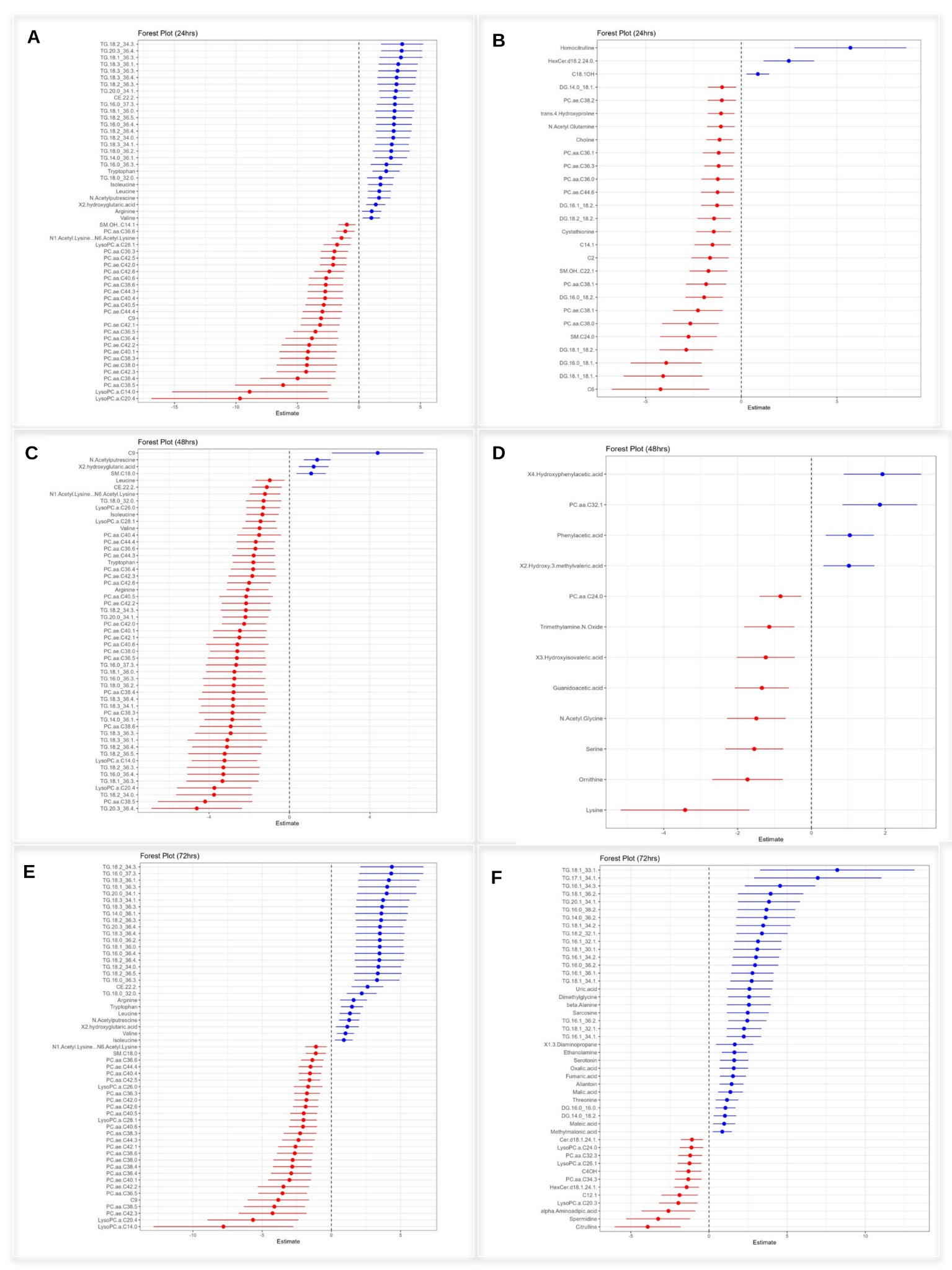

Supplement: Supplementary Figure S4 — Logistic-regression-based metabolite biomarkers of ARV infection. (A) Forest plot (24 h) showing the persistent upregulation of N-acetylputrescine and 2-hydroxyglutaric acid, with strong depletion of LysoPCs and PCs. (B) Forest plot (24 h) highlighting the stage-specific markers: homocitrulline (positive) and DG(18:1_18:1) (negative). (C) Forest plot (48 h) confirming the sustained elevation of N-acetylputrescine and 2-hydroxyglutaric acid with continued LysoPC/PC depletion. (D) Forest plot (48 h) showing lysine as the dominant negative marker and 4-hydroxyphenylacetic acid as the top positive predictor. (E) Forest plot (72 h) demonstrating persistent lipid remodeling with elevated N-acetylputrescine and 2-hydroxyglutaric acid and broad LysoPC/PC suppression. (F) Forest plot (72 h) highlighting stage-specific markers: strong TG(18:1_33:1) upregulation and citrulline depletion, consistent with impaired nitric oxide synthesis. [file Image4.jpeg]
